# Special Issue on Whole Genome Amplification

**DOI:** 10.3390/ijms24119626

**Published:** 2023-06-01

**Authors:** Richard Jäger

**Affiliations:** 1 Department of Natural Sciences, Bonn-Rhein-Sieg University of Applied Sciences, von-Liebig Str. 20, 53359 Rheinbach, Germany; richard.jaeger@h-brs.de; 2 Institute for Functional Gene Analytics, Bonn-Rhein-Sieg University of Applied Sciences, Grantham Allee 20, 53757 Sankt Augustin, Germany; 3 Institute of Safety and Security Research, Hochschule Bonn-Rhein-Sieg, University of Applied Sciences, Grantham Allee 20, 53757 Sankt Augustin, Germany

The development of whole-genome amplification (WGA) techniques has opened up new avenues for genetic analysis and genome research, in particular by facilitating the genome-wide analysis of few or even single copies of genomic DNA, such as from single cells (prokaryotic or eukaryotic) or virions. Using WGA, the few copies of genomic DNA obtained from such entities are unspecifically amplified using PCR or PCR-related processes in order to obtain higher DNA quantities that can then be successfully analysed further.

To achieve complete coverage of the genome of interest, overlapping or contiguous fragments are generated either through amplification using randomized primers capable of unspecifically hybridizing to all regions of the genomic DNA, or by splitting the DNA at random sites to generate a vast number of fragments that are fused to fixed DNA sequences for priming subsequent amplification. These two general principles are instantiated in different ways, and the various WGA methods thus established display specific strengths as well as shortcomings. Therefore, the choice of method requires thorough consideration in terms of suitability for a particular source of genomic DNA and type of downstream analysis.

A large area of the applications of WGA is the genetic analysis of single cells, often in biomedical contexts, such as single human parasites, single viral genomes, single disease-causing bacteria, single embryonic cells, and single cancer cells. More basic research questions that may benefit from single-cell WGA are, for example, the study of meiosis or of genetic alterations in cell lineages. Finally, WGA can assist in the analysis of samples with very low DNA amounts that may, however, derive from several cells, such as ancient DNA, environmental DNA, and DNA from forensic traces.

While being restricted to a small number of currently important fields of application, the six articles collected in this Special Issue complement each other well in terms of explaining and discussing the technical principles, the major challenges, and the perspectives of WGA. Thus, I am confident that the information provided will be relevant and helpful for researchers applying, or considering applying, WGA to a variety of appropriate scientific or analytical tasks.

Three articles in this Special Issue give overviews on the technical principles of the main WGA methods in the context of specific fields of application. In their article on WGA in preimplantation genetic testing (PGT), Volozonoka et al. [1] describe the principles of DOP-PCR, MDA, MALBAC, and PicoPlex and review their performance in terms of coverage, uniformity, and the generation of artifacts. The article by Jäger [2] explains the techniques thus far evaluated in forensic DNA profiling, among them the PCR-based methods DOP-PCR, PEP PCR, and adaptor ligation-mediated PCR, and MDA methods such as classical MDA and variants using circularized DNA substrates, the latter aiming at improving the analysis of fragmented DNA. Finally, Khan et al. [3] describe methods optimized for single-cell analysis. Apart from PCR-based methods and MDA variants, their article also covers the more recently developed methods, such as MALBAC, LIANTI, PTA, and META-CS, that aim to improve the uniformity and completeness of coverage, which are important parameters when analysing single circulating tumour cells (CTCs).

These three articles also highlight general and application-specific drawbacks of WGA and discuss the ability of particular WGA methods to overcome or avoid some of these drawbacks. In terms of general problems, amplification bias, incomplete coverage, and the generation of artifacts emerge.

In their article, Volozonoka et al. [1] give a comprehensive review on WGA in PGT. They thoroughly compare the performance of the different WGA methods in the context of PGT and discuss several established workflows that utilize different WGA methods in conjunction with massively parallel sequencing (MPS). As they report, both the WGA method and MPS parameters influence the WGA performance, and the review provides detailed information on the impact of these factors on specific analytical questions in PGT.

As discussed in the article by Jäger [2], in light of the particular challenges posed by forensic DNA profiling, all WGA methods tested thus far display serious limitations, such as the low quality of template DNA, the propensity of the analysed STR loci for replication slippage during PCR amplification, and the often non-uniform or incomplete coverage that may hamper the valid interpretation of DNA profiles. Nevertheless, as suggested in the article, in specific forensic applications, WGA may hold some promise.

Finally, Khan et al. [3] review the specific advantages and challenges of the different WGA methods in CTC analysis and inform on their respective suitability for various types of downstream analysis. In particular, the article contains useful information on studies that applied WGA to CTCs of specific cancer types, followed by analysis of their relevant diagnostic DNA markers.

A decisive factor for the performance of WGA protocols is the type of DNA polymerase used for amplification. Understanding the properties of DNA polymerases is instrumental for the improvement of existing methods and for the development of new methods. The article by Ordóñez and Redrejo-Rodríguez [4] contained in this Special Issue is the first review covering the types and properties of DNA polymerases specifically in relation to the performance of WGA. After explaining the most important WGA methods with special attention to the DNA polymerases employed, the authors review the characteristics of the different DNA polymerases available and how they impact parameters such as the fidelity, bias, and coverage of the different WGA methods. Importantly, the article addresses the ability of the DNA polymerases to function with damaged DNA, which is of relevance for several applications, such as the analysis of environmental DNA or ancient DNA, forensic DNA profiling, or the analysis of DNA from archival tissue specimens. Finally, a perspective is given on genetic engineering and molecular evolution approaches for improving the suitability of the DNA polymerases for WGA.

Two articles in this Special Issue report on original work, experimentally assessing the performance of WGA in the context of specific research questions.

In their article, Raz et al. [5] wished to identify the WGA method best suited for the reconstruction of cell lineages of single cells based on the identification of cumulating mutations. With this aim, the authors analysed clonally derived single cells and targeted a substantial number of STR loci through MPS using molecular inversion probes (MIPs). The study took advantage of the high proportion of heterozygous genotypes at STR loci, which allowed the researchers to determine the frequency of replication slippage artifacts, coverage, and allelic balance achieved with various single-cell WGA methods.

The article by Sobol and Kaster [6] addresses WGA-based genomics of single bacterial cells. Molecular crowding through volume reduction is proposed as a strategy to improve amplification success in samples with low DNA amounts. The article gives a general overview on microbial single-cell genomics using WGA and on volume reduction techniques based on microfluidics or other nanolitre methods. Using acoustic liquid dispensing technology, the authors investigate the relationship between the reaction volumes and amplification bias of multiple displacement amplification (MDA) applied to single bacterial cells and show that uniformity and coverage can be optimized by adjusting reaction volumes in the microlitre range.

The articles assembled in this Special Issue show that in some areas, such as CTC analysis, microbiome analysis, and PGT, WGA methods are viable options for genetically characterizing few or single cells. In forensics, which would appear to be a most natural field of application in light of the often low amounts of trace DNA, the principal drawbacks of WGA likely limit its applicability to only a very narrow range of sample types, and reliable usage in casework still awaits being demonstrated. This Special Issue thus highlights the prospects and the current limitations of WGA and will hopefully stimulate future research applying WGA or improving its performance and extending its range of applications.
